# Surface Engineering of Nanomaterials with Polymers, Biomolecules, and Small Ligands for Nanomedicine

**DOI:** 10.3390/ma15093251

**Published:** 2022-04-30

**Authors:** Ana M. Díez-Pascual

**Affiliations:** Universidad de Alcalá, Facultad de Ciencias, Departamento de Química Analítica, Química Física e Ingeniería Química, Ctra. Madrid-Barcelona, Km. 33.6, 28805 Alcalá de Henares, Madrid, Spain; am.diez@uah.es

**Keywords:** functional nanomaterials, nanomedicine, polymers, tissue engineering, cancer therapy, drug delivery, medical implants

## Abstract

Nanomedicine is a speedily growing area of medical research that is focused on developing nanomaterials for the prevention, diagnosis, and treatment of diseases. Nanomaterials with unique physicochemical properties have recently attracted a lot of attention since they offer a lot of potential in biomedical research. Novel generations of engineered nanostructures, also known as designed and functionalized nanomaterials, have opened up new possibilities in the applications of biomedical approaches such as biological imaging, biomolecular sensing, medical devices, drug delivery, and therapy. Polymers, natural biomolecules, or synthetic ligands can interact physically or chemically with nanomaterials to functionalize them for targeted uses. This paper reviews current research in nanotechnology, with a focus on nanomaterial functionalization for medical applications. Firstly, a brief overview of the different types of nanomaterials and the strategies for their surface functionalization is offered. Secondly, different types of functionalized nanomaterials are reviewed. Then, their potential cytotoxicity and cost-effectiveness are discussed. Finally, their use in diverse fields is examined in detail, including cancer treatment, tissue engineering, drug/gene delivery, and medical implants.

## 1. Introduction

Nanomaterials with unique physicochemical properties have recently attracted a lot of attention since they offer a lot of potential in many fields, particularly in biomedical sciences, including drug and gene delivery systems [1,2,3], cancer treatment [4,5], monitoring systems [6], tissue engineering [7], and so forth. New generations of engineered nanostructures, also known as designed and functionalized nanomaterials, have opened up new possibilities in the applications of biomedical approaches such as biomolecular sensing, drug delivery, biological imaging, and therapy. A wide number of nanomaterials have great potential to be used in biomedicine, including nanotubes, nanoparticles, nanoplates, and nanowires, to mention but a few [8,9,10]. Nonetheless, they must meet specific characteristics to be used in biomedical applications [11]. In this regard, their potential cytotoxicity, which can be induced by their structure, chemical content, or features, for example, as well as their biocompatibility, have to be assessed [12]. Their colloidal stability should also be maintained under physiological conditions, ideally across a wide pH range [13]. As a result, it is critical to consider these criteria to ensure the safety, nontoxicity, and biocompatibility of the nanomaterials. Specific interactions with polymers, natural biomolecules, and synthetic ligands of interest are required to modify and functionalize the nanomaterial surface in order to meet these criteria [14,15,16].

The methods for creating and manipulating functionalized nanomaterials (FNMs) open up exciting new opportunities for developing novel multifunctional biological devices [17]. Furthermore, functionalization prevents nanoparticles from agglomeration and makes them compatible in subsequent phases. As a result, FNMs can transport more efficiently after systemic injection and have better pharmacokinetic characteristics in vivo. FNMs can be deeply driven into tissues through narrow capillaries and epithelial coating, leading to improved therapeutic agent delivery to the targeted location [18]. Furthermore, the small size of FNMs enhances exceptional physicochemical features such as solubility, diffusivity, immunogenicity, and the capacity to target the designated region with minimum diffusion to its surrounding [19,20].

The nanomaterial interface can be designed and applied in different ways. These approaches are classified as replacement, noncovalent, and covalent conjugations based on the primary concept of the type of functionalization interaction [21]. The interface between nanoparticles (NPs) and the attached molecules is modified via the replacement approach, which comprises ligand exchange and ligand addition [22]. Noncovalent techniques rely on many interactions, most of them weak, such as electrostatic, Van der Waals, hydrophobic, and hydrogen bonds, and it is particularly useful with metallic nanoparticles [23]. They are straightforward and do not modify the molecular structure nor their interaction with targets. However, these modifications are strongly dependent on parameters such as ionic strength and pH. On the other hand, covalent attachment techniques have been proposed to alter the external functionalization of nanomaterials to bind molecular entities for biomedical purposes, hence giving the nanoparticles additional functionality [24]. 

**Figure 1:**
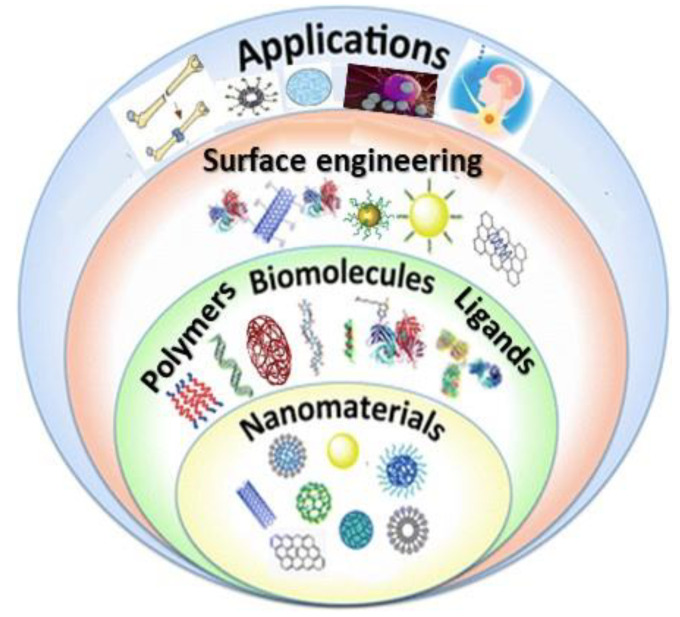
Schematic representation of the functionalization of different types of nanomaterials by polymers, natural biomolecules, and synthetic ligands, and their applications in nanomedicine.

This study aims to provide particular examples to cover the different ways of nanomaterial functionalization using polymers, natural biomolecules, and small ligands (Figure 1), via covalent and noncovalent conjugation. Before highlighting specific examples of each type of functionalization, the basis of the functionalization will be summarized. Although some studies on nanoparticle surface modification for medical and nanotechnological application have been reported [8,10,11,12,25], most of them are not updated, deal only with nanoparticles rather than nanomaterials in general, and focus only on either the nanoparticle synthesis or on certain biomedical applications. Thus, the current paper reviews recent studies on nanomaterial surface engineering, divided by nanomaterial type and specialized uses. Besides, the cytotoxicity, cost effectiveness, and use of FNMs as a versatile tool in nanomedicine will be discussed. Due to their beneficial characteristics such as biodegradability and biocompatibility in physiological mechanisms, wide availability, suitability for chemical treatment, and wide range of potential synthesis process from different sources, nanomaterials have been extensively explored in the literature. This article offers novel insights on surface functionalization of nanomaterials, focusing on their therapeutic, diagnostic, tissue-engineering, and medical-implant applications. Following a brief overview of the different surface modification strategies and different types of functionalized nanomaterials, a summary of the most relevant biomedical applications is presented.

## 2. Strategies for Surface Functionalization of Nanomaterials

**Figure 2:**
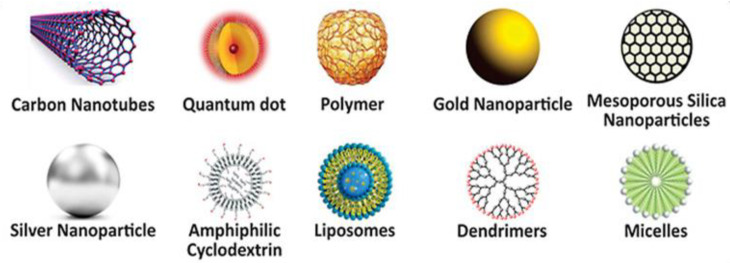
Representation of different types of organic and inorganic nanomaterials used in nanomedicine.

The exclusive properties of nanomaterials compared to their microsized counterparts, such as big, specific surface areas and nanometer sizes, have involved huge attention in the scientific community. Depending on the desired final properties, the composition of nanomaterials can vary from metals or metal oxides to carbon or polymers (Figure 2). Metallic nanoparticles (like gold or silver) are beneficial for designing drug delivery and imaging systems, but their safety has to be investigated in detail to prevent undesirable side effects in humans [26]. Iron oxide, with outstanding magnetic properties, is the most common selection as the core of functionalized nanoparticles. Silica NPs are frequently used in drug delivery applications. Mesoporous silica nanoparticles (MSN), with tunable pore size, are widely used to load small molecules, including amino acids, nucleic acids, drugs, and so forth [27]. However, due to reactive surface silanol groups, there are biocompatibility issues regarding the use of silica nanoparticles for nanomedicine. Concerns regarding the toxicity of carbon-based nanomaterials such as carbon nanotubes (CNTs), quantum dots (QDs), and graphene have also been reported [28]. Polymeric nanoparticles are a widespread option for biomedical applications owed to their tailorable physicochemical properties, excellent biocompatibility, and capability to liberate molecules in a continued way [29]. Numerous polymeric micelles such as thiomers, pluronic, polysaccharides, and polyethylene glycol (PEG) have been investigated [14]. In addition, other colloidal nanostructures such as dendrimers, liposomes, polysomes, and cyclodextrins have been designed for targeted applications [30]. 

**Figure 3:**
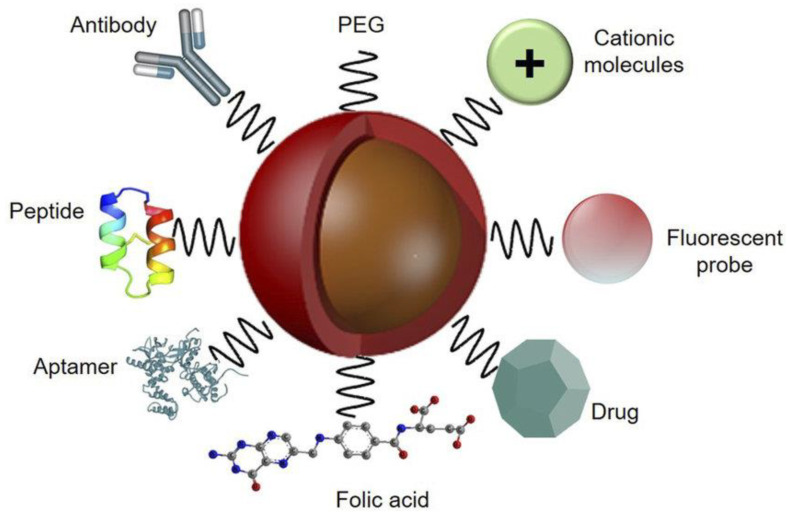
Representation of a nanoparticle functionalized with different types of ligands, polymers, therapeutic compounds, and biomolecules. Adapted from Ref. [40], copyright 2020, with permission from Impact Journals LLC.

Various types of targeting agents have been implemented to be incorporated on the surface of nanoparticles, especially peptides [31], aptamers [32], antibodies [33], polyethylene glycol (PEG) [34], cationic molecules, folic acid [35], drugs, and fluorescent probes, as depicted in Figure 3. It should be noted that biomolecular interactions rely on the chemical modification of the nanoparticle surface when using NPs for in vitro or in vivo applications [36]. Through a ligand–receptor interaction, such targeting moieties can allow nanoparticles to be embodied into cancer cells and tissues. To facilitate active targeting of NPs to receptors, which are located on the surface of the membrane, the nanoparticle surface can be tailored with targeting ligands, resulting in increased cellular internalization and/or selective absorption via receptor-mediated endocytosis [37]. Researchers are particularly interested in discovering new biomarkers and their relevant ligands in targeted medication administration. The binding of NPs to analytes, pathogens, and biomarkers might cause their signal to be amplified, making it easier to detect and image [38]. When the scaffold surface is decorated with bioactive cues to allow FNPs to interact with cells and the extracellular matrix (ECM) to elicit tissue-specific phenotypes, this is referred to as functionalization [39]. Chemists can easily make the suitable functionalities for use in clinics thanks to the easiness of such functionalization. For example, cell surface molecules have been used to identify nanoparticles functionalized with ligands that show a varied affinity for proteins [34].

Furthermore, functionalization has been proven to protect NPs against agglomeration and make them biocompatible materials in other application stages [41]. Functionalization improves the NPs’ physical, chemical, and mechanical characteristics, resulting in synergetic effects [42].

### 2.1. Functionalization by Covalent Conjugation

**Figure 4:**
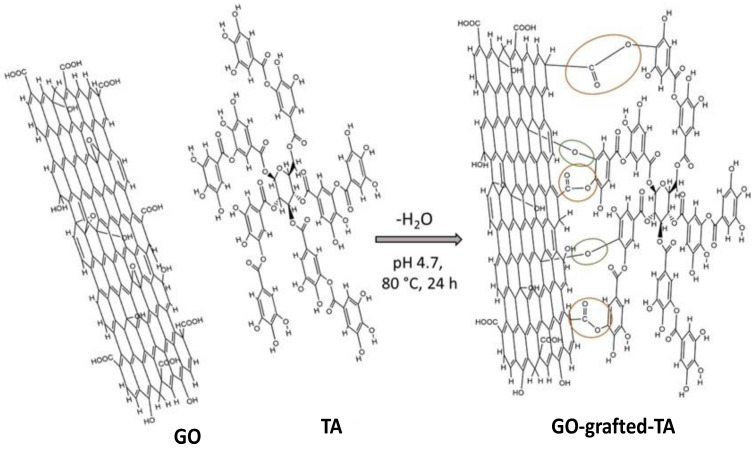
Representation of the covalent functionalization of graphene oxide (GO) with a biological macromolecule, tannic acid (TA) via formation of ether and ester linkages. Reproduced from Ref. [45], copyright 2022, with permission from Elsevier.

The covalent conjugation comprises the reaction of a conjugator (also named linker) with a certain species or chemical group, in a way that the molecules are attached on the nanomaterial surface [43]. Carboxylic acids, amines, thiols, disulfides, phosphates, nitriles, and so forth have been used for covalent conjugation via chemical reactions [44]. Amine groups are the most widely used for functionalization in the biomedical field. The strategy consists in anchoring small molecules or proteins on the nanoparticles. Further, amine functionalization can be used with the aid of n-hydroxysuccinimide (NHS) and different carbodiimides such as EDC. Similarly, carboxylic groups can form ester or amide bonds with alcohol or amine groups on the NPs’ surface [45], Figure 4. On the other hand, conjugation on metallic NPs can be effectively carried out via the thiol moiety. The interaction occurs by reaction of sulfhydryl (RSH) groups on the metallic nanoparticles.

### 2.2. Functionalization by Noncovalent Conjugation

The noncovalent bonding comprises the attachment of molecules on the surface of nanomaterials without chemical bonding via physical adsorption and/or wrapping of molecules by weak interactions such as hydrophobic (Van der Waals), H-bonding, cation−π, anion−π, π–π, and H−π, that preserve the intrinsic properties of the nanomaterial [43]. This approach has some benefits over the covalent way: (i) it takes place under moderate conditions (water solution at room temperature), thus avoiding structural damage of the nanomaterial; and (ii) enables control of the amount of adsorbed/wrapped molecule. The versatility of this route enables a large number of substances to be coupled to the nanomaterials including polymers, solvents, surfactants, aromatic compounds, etc. In order to offer steric stabilization, nanomaterials have been anchored to biocompatible polymers such as polyethylene glycol (PEG) [46]. 

### 2.3. Functionalization by Biomolecules

Biomolecules are outstanding candidates to apply in the surface engineering of nanoparticles. Biomolecule-coated nanoparticles have features that are troublesome or inconceivable to attain with synthetic materials, such as excellent bio-macromolecule distribution with little cytotoxicity. Biomolecules such as proteins, peptides, antibodies, and oligonucleotides can be very valuable for targeting NPs to cancer cells where particular receptors are overexpressed. The synthesis of gold–thiol bonds to create oligonucleotide–AuNP conjugates was one of the first bio-nanotechnology examples reported in the literature [34]. Proteins or peptides boost the penetration of NPs into cells via receptor-mediated endocytosis. On the other hand, transferrin is a glycoprotein that can bind to specific receptors on the cell membrane. A few articles have reported the benefits of using this protein as a target for Au, MSN, and poly(lactic-co-glycolic acid) (PLGA) nanoparticles [30]. 

**Figure 5:**
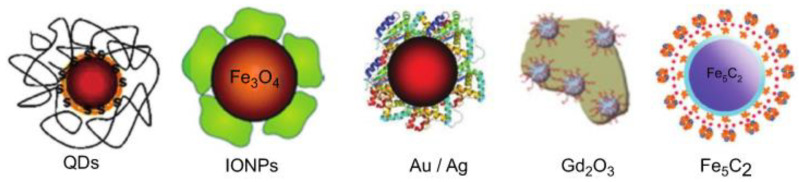
Representation of the surface modification of different types of NPs with albumin. Reproduced from Ref. [47], copyright 2016, with permission from John Wiley and Sons.

Albumins are a class of naturally occurring proteins that, besides being applied to load imaging and therapeutic agents, are valuable for modification of numerous types of NPs, as depicted in Figure 5 [47]. Surface modification of NPs with albumins, such as bovine serum albumin (BSA), provides higher water solubility, increased biocompatibility and blood circulation time, and improved stability and cellular interactions compared with uncoated nanoparticles. Different strategies for conjugation of NPs such as AuNPs with albumin have been reported [48], including: (1) Passive adsorption, so that the charged groups of the protein are anchored to the NP surface via covalent or noncovalent interactions. (2) Active adsorption, which involves the use of modified albumin in order to strengthen the albumin-NP interactions. (3) The use of this protein for NP synthesis, either as a reagent (i.e., reducing agent), foaming, stabilizer, or building block for NP synthesis, resulting in NPs with an albumin coating [49]. The use of albumin encapsulation methods provides some profits, such as the loading of agents with low solubility in order to protect them from degradation. (4) Desolvation cross-linking (coacervation process), used to produce core–shell albumin-NPs. This strategy allows chemical agents to become trapped within albumin capsules, which are very stable and protect from degradation. (5) Emulsification: an albumin solution and a nonaqueous phase are mixed, giving rise to an emulsion, and the NP is dissolved in the oil phase. This methodology is used for the encapsulation of lipophilic drugs and enhances aqueous solubility and biocompatibility. (6) Thermal gelation: an albumin water solution is heated to induce protein unfolding, which results in protein–protein interactions by disulfide and hydrogen bonding, as well as hydrophobic and electrostatic forces. Besides, unfolding induces NP–protein interactions, leading to a protein coating onto the NP surface.

### 2.4. Functionalization by Polymers

**Figure 6:**
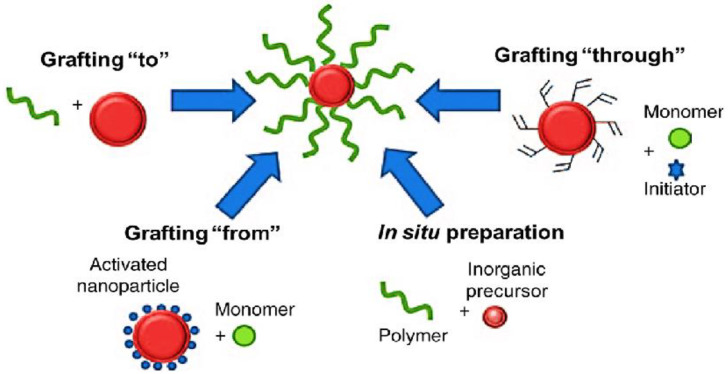
Schematic illustration of polymer grafting approaches: “grafting from”, “grafting to”, “grafting through” and “in situ” preparation in the presence of an inorganic precursor.

A large number of biocompatible, commercially accessible polymers can be used for functionalization, and are typically chosen based on their specific properties such as hydrophobicity, melting point, and functional groups. Polymers frequently used as NP coatings comprise synthetic polymers (i.e., PEG [50] and PLGA [51]) and natural polymers (such as chitosan [52,53]). Polymers have been used for both covalent and noncovalent conjugation of a wide range of nanomaterials. The covalent approach involves the “grafting” (chemical anchoring) of polymeric segments to the NM surface, and can be implemented via “grafting to”, “grafting from”, “grafing through” and “in situ” tactics (Figure 6). The former is based on the synthesis of a modified polymer prone to react with the functional groups on the surface of the nanomaterial [46]. A shortcoming of this tactic is that the amount of polymer grafted to the nanomaterial is restricted, owed to the low reactivity and large steric barrier of the polymeric segments. In the “grafting from” path the polymer is grown from the NM surface via polymerization of monomers [43]. This approach is effective and manageable, owed to the high reactivity of monomers, allowing a high grafting level. A variation of this strategy is to carry it out via “in situ” polymerization in the presence of the inorganic precursor. Nonetheless, this method requests precise monitor of the amounts of each reagent and the polymerization conditions. In the “grafting through”, a low molecular weight monomer is radically copolymerized with a polymerizable macromonomer in the presence of an initiator. 

Polymers are suitable for functionalization because they create a physical barrier around the NPs, preventing the core of the NPs from coming into direct contact with biological receptors. Polymers can produce a physical barrier but with a reduced hydrodynamic radius. As a result, polymer coatings outperform small molecule ligands when imparting macromolecular system characteristics to the particle surface, similar to biological proteins. The use of polymers such as PEG to coat nanoparticles improves passive tumor tissue targeting, increasing permeability, and retention (EPR), as well as biocompatibility [54]. This PEG and other polymer coatings decrease blood serum protein adsorption, lengthen circulation duration, and promote particle absorption into tumor tissues [34]. Using AuNPs synthesized by stacking cationic polyallylamine and anionic poly (acrylic acid) polyelectrolyte layers, Kleinfeldt and coworkers [55] developed an excessively hydrophilic and biocompatible coating that enables colloidal stability. Makvandi et al. [56] investigated the functionalization of various polymers (glyclusters, glydendrimers, glycopolymers) and nanomaterials (Ag_2_O, CuO, ZnO, Fe_3_O_4_, MgO, TiO_2_, Se, Ni, Pd) for water purification, food containers, fabrics, and medical applications. The benefits and drawbacks of polymer functionalization were investigated and explored in that study. When natural or synthetic polymers are used to functionalize NPs, photo/thermo-responsive properties can be achieved [57]. For instance, chitosan grafted with poly-L-lactide using thiourea-functionalized, and poly-N-isopropyl acrylamide were used to synthesize photo/thermo-triggered micelles [58].

### 2.5. Functionalization by Small Ligands

Small ligands are a common selection for functionalizing nanomaterial since they are relatively simple to chemically bond to surfaces via functional moieties in their structure. They are an appropriate choice to adjust the nanomaterial properties such as hydrophilicity or charge with a view to improve their biological activity and interaction with other biologically essential ligands, as well as their stability, aqueous solubility, drug loading, and so forth. For instance, silica NPs can be straightforwardly tailored with organosilane molecules such as 3-(aminopropyl) triethoxysilane (APTES) through silane chemistry. It has been reported that APTES-functionalization is an effective method for adjusting drug loading and discharge from mesoporous silica nanoparticles (MSN) [59]. Besides, it is beneficial for many aims, such as the release of low soluble drugs, the targeting of drugs to a chosen position, or to make multifunctional drug delivery and imaging devices. Other ligands such as drugs have been used for tailoring NP surfaces. For example, doxorubicin (DOX, a frequently applied anticancer drug) has been conjugated to Fe_3_O_4_ NPs with the aim to develop dual-functional NPs [60]. These modified NPs can destroy tumor cells via the conjugated DOX, and concurrently enable magnetic resonance imaging of the tumor, which is highly valuable. Other small drugs such as methotrexate, that can target the folate receptor on cancer cells, ciprofloxacin [25,61], and so forth, have been conjugated with different nanomaterials.

Various nanomaterials functionalized by small ligands can be added as signal reporters or as carriers for loading more signal reporters in biosensors for analyte detection [62]. Mahmoudpour et al. [63] designed a method for producing aptameric functionalized materials (AFMs). Optical indicators, conducting transducers, carriers, catalysts, and other features, were combined to develop advanced AFMs. Drug delivery, bioimaging, and appropriate sensing have been highlighted as biological uses of improved AFMs. Aptamers have been identified among the most promising prospects for constructing a broad range of sensing platforms due to their unique properties, such as outstanding specificity and sensitivity, easiness of fabrication, and excellent durability in a variety of circumstances. For the manufacturing of aptamer-based nanoprobes, many signals-transduction approaches have been developed. Incorporating numerous aptasensing techniques with NPs has improved biosensor selectivity and sensitivity in recent years [64].

## 3. Functionalized Nanomaterials

### 3.1. Metallic Nanoparticles

**Figure 7:**
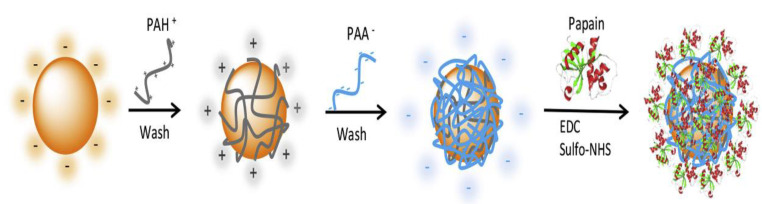
Schematic representation of the functionalization process of AuNPs with papain. The surface modification was achieved by a layer-by-layer (LbL) approach via activation of COOH groups of the modified AuNPs with EDC and sulfonated NHS as coupling agents, followed by amide bonding with the NH_2_ groups of papain. (PAH+, polyallylamine hydrochloride; PAA−, polyacrylic acid sodium). Adapted from Ref. [68], copyright 2017, with permission from Elsevier.

Metal-based nanomaterials consist of nanoparticles of raw metal, such as gold (Au) and silver (Ag). AuNPs are inert in bulk, while become highly reactive in nanoparticle form [65]. The exciting surface chemistry of AuNPs opens up novel routes for the progress of unexplored multifunctional instruments for biomedical and nanotechnological applications [66]. Nanotechnology applications have drawn a great deal of interest since the late 1980s [67]. The exceptional electrical and optical properties of Au boost their use in biosensing and bioimaging. The use of organic molecules to functionalize Au NPs aids the conjugation of drugs for delivery systems. Thus, AuNPs can be used as photothermal therapeutic agents [56]. For instance, surface-modified AuNPs have been prepared via a layer-by-layer procedure with alternating polyelectrolyte layers of cationic polyallylamine and anionic poly(acrylic acid). Subsequently, papain was covalently immobilized on the modified AuNPs via amide bond between the NH_2_ groups of papain and the terminal COOH groups of the modified NPs, using EDC and sulfonated NHS as coupling agents, as depicted in Figure 7, to produce a heterogeneous biocatalyst that has been applied in bioanalysis and biopharmaceutical analysis [68].

**Figure 8:**
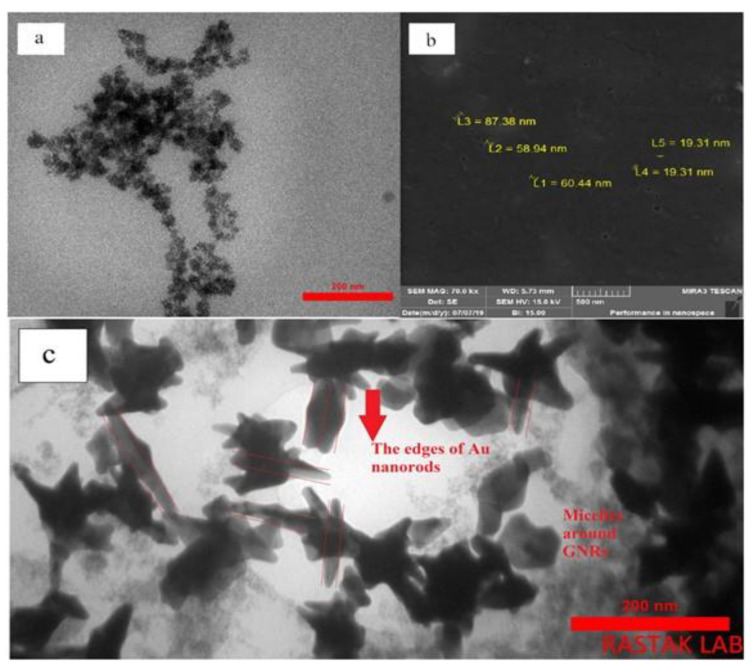
(**a**) TEM image of polymeric micelles, (**b**) SEM image of AuNRs, and (**c**) TEM image of AuNRs coated by polymeric micelles. Adapted from Ref. [69], copyright 2020, with permission from Elsevier.

The conjugation of gold nanorods (AuNRs) onto micelles, via gold-thiolate complex formation, brings photosensitivity to the nanoassembly. The size and surface morphology characterization via TEM (Figure 8) indicated that the mean micellar size was around 15 nm, and the thickness and length of the AuNRs was about 20 and 65 nm, respectively. The percentage of conjugated AuNRs to the micelles was roughly 12%. The attachment of chitosan transfers the photosensitivity of functionalized AuNRs to micelles, and the micelle thermal shrinkage induces the release of paclitaxel, a drug widely used to treat breast cancer [69].

**Figure 9:**
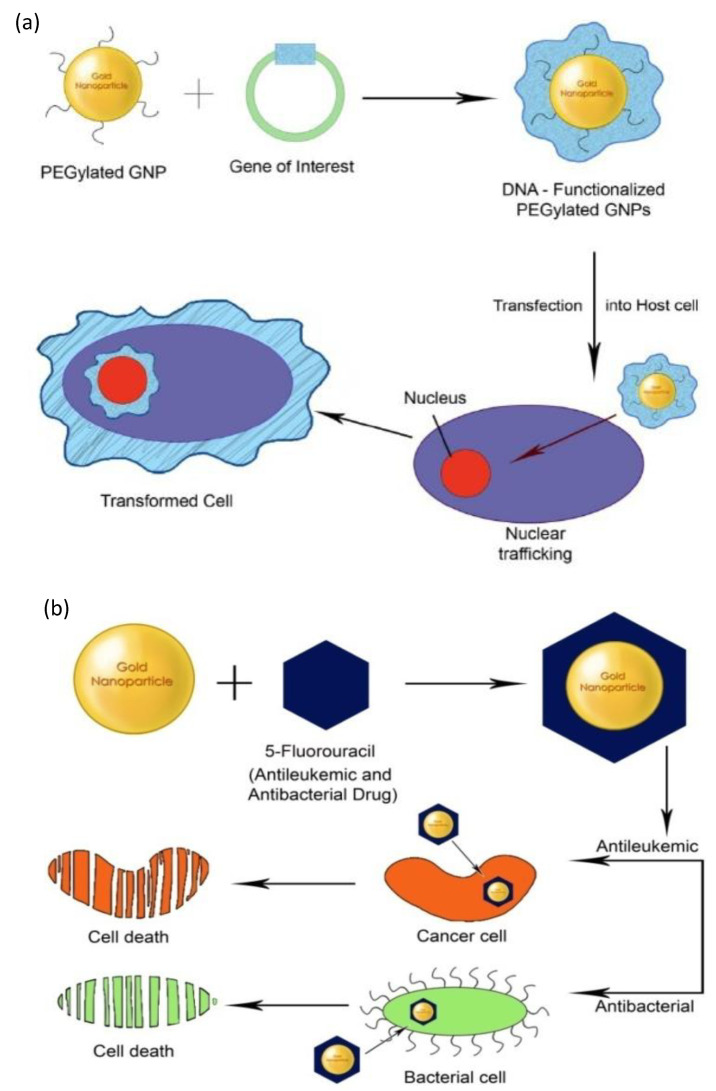
(**a**) PEGylated gold nanoparticles for gene delivery. (**b**) Functionalized gold nanoparticles for drug delivery. Adapted from Ref. [70], copyright 2011, with permission from MDPI.

Nejati et al. [19] examined functionalized AuNPs in biomedical applications. To attain this goal, their structure, production, and functionalization were extensively explored and discussed. Gold NPs have been utilized in biological applications, electrochemical technology, and radiation oncology. Multifunctionalization, that is, functionalization that allows for the provision of more than one attribute at a time, provides added value to these NPs due to synergistic effects. Multifunctionalized gold NPs have been discovered to be a viable choice in biomedicine for delivering anticancer drugs and antibiotics for combined photothermal and chemical therapy [70]. AuNPs are suitable for the delivery of the drugs to cellular destinations due to their ease of synthesis, functionalization, and biocompatibility. Figure 9 depicts functionalization of AuNPs for gene and drug delivery. AuNPs functionalized with targeted particular biomolecules can successfully kill tumor cells or bacteria (Figure 9). Large surface-to-volume ratio of AuNPs can carry a huge amount of drug molecules. AuNPs have been applied for the codispensation of protein drugs owed to their skill in penetrating cell membranes, probably because they can interact with the lipids present on the cell surface [70].

Despite the efforts carried out, more studies into intelligent drug delivery based on nanoparticles, particularly gold NPs, is required. Despite numerous publications, only a few clinically authorized drug delivery nano systems are currently accessible in the industry. As a result, an immediate need is found to incorporate animal-model research into clinical practice [70]. Donoso–Gonzalez et al. [71] used cationic cyclodextrin-based polymer (CCD/P) to load phenylethylamine (PhEA) and piperine (PIP) onto gold nanostars (AuNSs). They evaluated the product potential for simultaneous drug loading and SERS-based detection. In addition to PhEA and PIP, the polymer contained AuNSs that had been functionalized with PhEA and PIP, resulting in a unique AuNS-CCD/P-PHEA-PIP nanosystem with an optimum size and Z potential for biomedical applications. Hybrid materials incorporating carbon nanomaterials and AuNPs have also been synthesized. For instance, Shon et al. [72] reported the synthesis of soluble fullerene-linked AuNPs using a modified Brust reaction and subsequent ligand exchange reaction of hexanethiolate-protected Au NPs with 4-aminothiophenol. Amination of C60 with 4-aminothiophenoxide ligands produced the C60-linked AuNPs. This approach enables the control of the optical and photochemical properties of the nanoparticles. Sudeep and coworkers [73] developed a self-assembled photoactive system comprising AuNPs as the central core and fullerene moieties as the photoreceptive hydrophobic shell via functionalization of the NPs with a thiolated fullerene derivative. Yaseen et al. [74] used C60-terminated alkanethiol to synthesize novel fullerenethiol-functionalized gold nanoparticles (C60−AuNPs) of 2 nm diameter with an extremely narrow size distribution. The fullerene-thiol moiety was inserted into the fullerene by the ligand exchange method. Liz–Marzán et al. [75] developed Au core/SiO_2_ shell nanocomposites with tailorable thickness and good dimensional stability. Citrate-capped AuNPs were first synthesized and then reacted with aminopropyl trimethoxysilane, a widely used coupling agent, which anchored onto the NPs via silanol groups. Active silica was subsequently added, leading to the formation of a fine, dense, and fairly homogeneous silica layer wrapping the NPs.

On the other hand, AgNPs are antibacterial and anti-inflammatory, and possess excellent biocompatibility [76]. The AgNPs can be straightforwardly synthesized via simple, fast, nontoxic, and environmentally friendly means so that they can produce NPs with perfectly defined morphology and size. They were applied as a coating for cardiovascular implants to improve their biocompatibility. Additionally, their antimicrobial, antifungal, antiviral antiangiogenic, and anticancer properties make them suitable in a large number of biomedical and health care areas including device coatings, drug delivery systems, wound dressings, the textile industry, photothermal therapy, and so forth. The biological activity of AgNPs is influenced by many parameters such as the NP shape, size, morphology, state of dispersion, solution rate, reactivity, and ion discharge efficiency, amongst others, which condition their cytotoxicity. The design of AgNPs with uniform functionality, size, and morphology is crucial from a practical viewpoint. Other metallic NPs such as ruthenium and selenium have been applied in nanomedicine [77], in particular for drug delivery. 

### 3.2. Metal Oxide-Based Nanomaterials

A wide number of variations of metal oxide NPs have been used in nanomedicine, such as iron oxide (Fe_2_O_3_, Fe_3_O_4_), CeO_2_, titania (TiO_2_), ZnO, NiO, silica (SiO_2_), and so forth [78,79,80]. Iron oxide NPs are a fascinating family of nanostructures that have attracted much interest in the medical area because of their negligible toxicity, high biocompatibility, and inherent magnetic properties, which make them perfect candidates for therapeutic and diagnostic goals, particle imaging, and as contrast agents in magnetic resonance imaging (MRI) and ultrasonic techniques [81]. The incorporation of Fe_3_O_4_ NPs also enhances the antimicrobial properties [82].

**Figure 10:**
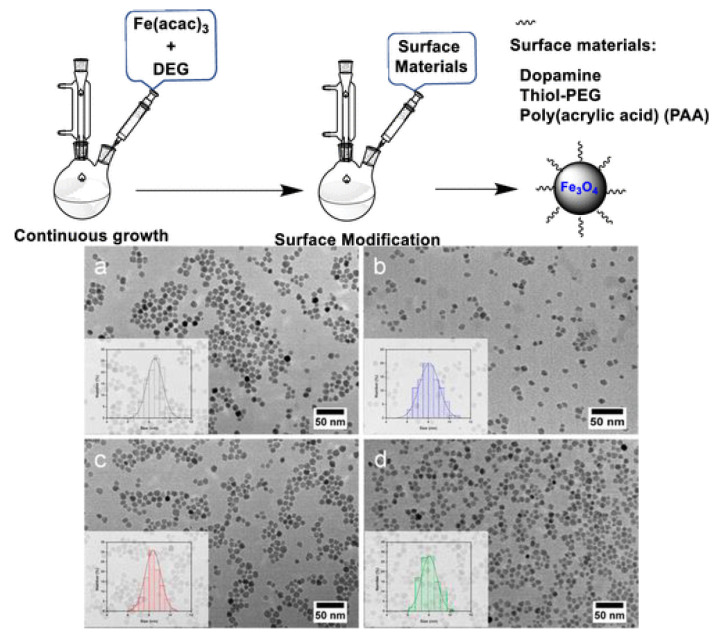
(**Top**) Synthesis of Fe_3_O_4_ NPs in diethylene glycol (DEG) by thermal decomposition of acetylacetonate (Fe(acac)_3_), and surface modification by adding surface ligands. (**Bottom**) TEM images of Fe_3_O_4_ NPs (**a**), Fe_3_O_4_ NPs functionalized with dopamine (**b**), Fe_3_O_4_ NPs surface modified with polyethylene glycol with thiol end group (thiol-PEG) (**c**), and Fe_3_O_4_ NPs modified with poly(acrylic acid) (PAA) (**d**). Adapted from Ref. [84], copyright 2021, with permission from the American Chemical Society.

The five most popular strategies to generate hollow iron oxide NPs are the Kirkendall effect, galvanic substitution, chemical etching, nano-template-mediated, and hydrothermal/solvothermal routes [83]. Cheah et al. [84] synthesized iron oxide NPs in diethylene glycol (DEG) by thermal decomposition of iron (III) acetylacetonate (Fe(acac)_3_), and subsequently changed the surface of the NPs by adding surface ligands (Figure 10). Using this easy production process, surface modification of iron oxide NPs with various covering substances such as dopamine (DOPA), polyethylene glycol with thiol end group (thiol-PEG), and poly(acrylic acid) (PAA) is achievable. The size of these NPs can be precisely controlled at the nanometer scale by continuous growth. TEM images confirmed that the morphology did not change upon functionalization (Figure 10). Besides, NPs with PAA coating can be used as contrast agents. The surface change of oleic-acid-coated iron oxide NPs (Fe_3_O_4_-OA) (made by coprecipitation method) with tetraethylorthosilicate was studied by Nayeem et al. [85] using an inverse microemulsion approach (TEOS). To obtain thermally sensitive magnetic nanocomposites (MNCs), Fe_3_O_4_/SiO_2_/P(NIPAm-co-AMPTMA), the surface of iron oxide nanoparticles was tailored using a multistep approach with poly [N-isopropylacrylamide-co-(3-acrylamidopropyl) trimethylammonium chloride], P(NIPAm-co-AMPTMA). Magnetic nanoparticles (MNPs) have been extensively studied as MRI contrast agents to aid in the detection, diagnosis, and treatment of solid cancers. The absorption of superparamagnetic iron oxide NPs (SPIONs) in the endothelial reticulum system (RES) can be used in medical imaging to detect liver neoplasms and metastases. It can also currently differentiate tiny lesions of 2–3 mm. Furthermore, ultrasmall superparamagnetic iron oxide NPs (USPIONs) show promising utility in MRI exams for the identification of lymph node metastases that are 5–10 mm wide [86]. By utilizing the distinct molecular fingerprints of these disorders, the future iteration of active targeting MNPs, which has recently been explored, has the capacity to enhance tumor detection and characterization [86].

Cerium oxide (CeO_2_) NPs, named as nanoceria, have the unique property of anti-inflammation. They have better redox as well as potential antioxidant properties with therapeutic characteristics. TiO_2_ has the unique properties of high chemical stability, cytocompatibility, and optical properties [87]. The biocompatible properties of TiO_2_ NPs have increased their usage in drug delivery, bone substitute materials, bone regeneration, cell and tissue behavior modulation, vascular stents, scaffolds, bioimaging, and biosensors [78]. MSN also have great potential for nanomedicine. In fact, upon functionalization, they can be efficiently targeted to cancer cells [59] and be used for encapsulation and controlled release of drugs [27]. For biomedical applications, ZnO possesses the properties of low toxicity and biodegradability. It can be used for the purpose of drug delivery, gene delivery, biosensing, bioimaging, etc. [88]. CuO NPs have also been used for targeted drug delivery in breast cancer therapy [35].

### 3.3. Ceramic-Based Nanomaterials

A wide range of ceramics, including Ca_3_(PO_4_)_2_, bioactive glass, Al_2_O_3_, ZrO_2_, CaCO_3_ and so forth, are getting countless interest in the biomedical field, particularly in the tissue engineering arena. Thus, their outstanding osteoconductivity, resorbability, biocompatibility, biodegradability, and hydrophilicity make them appropriate for numerous hard tissue applications [89,90]. They can be divided into three types: bioinert, bioactive, and resorbable. Resorbable ceramics are progressively adsorbed and substituted by endogenous tissue. They can be synthesized in the forms of nanocrystals, NPs, nanopowders, or nanocoatings. The most popular is Ca_3_(PO_4_)_2_, which is widely applied in the form of NPs and nanocements for orthopaedic and dental uses. The optimal surface charge density, functionality, and solution characteristics of this ceramic account for its fittingness in drug delivery and growth factor uses. Bioactive ceramics such as hydroxyapatite (HDA) NPs are a type of calcium phosphates that have been comprehensively investigated in bone regeneration and antibacterial applications [91,92]. They are osteoconductive and can link to bone tissues via chemical bonding, following the rule of bonding osteogenesis. Furthermore, for bone tissue engineering, bioactive glass is crucial, owed to its outstanding osteoconductivity, osteoinductivity, and biocompatibility [93]. Bioinert bioceramics such as ZrO_2_ have great chemical stability and in vivo mechanical strength. This oxide is regarded as a nontoxic material and has strong resistance to acids; hence, it is widely used in coatings for metallic load-bearing implants and dentistry. Another widely used oxide is Al_2_O_3_, which possesses high hardness and superior heat resistance, and has been applied in arthroplasty, dentistry, and as an antimicrobial coating [94].

### 3.4. Carbon-Based Nanomaterials

**Figure 11:**
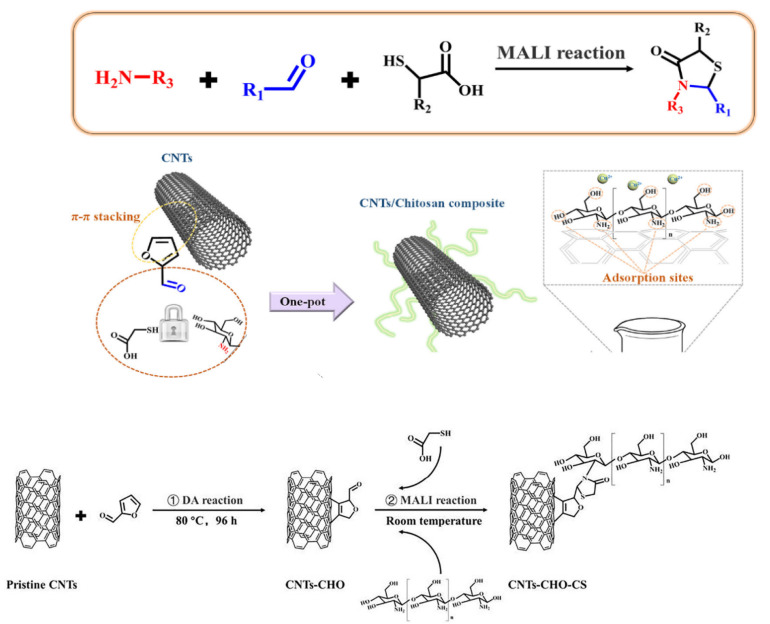
Functionalization of carbon nanotubes with chitosan based on MALI reaction. Adapted from Ref. [100], copyright 2019, with permission from Elsevier.

Within carbon-based nanomaterials, carbon nanotubes (CNTs), graphene oxide (GO), and graphene quantum dots (GQDs) have been broadly explored in biomedical applications [13,95,96]. Purification, separation, dispersion, stability, alignment, functionalization, and arrangement of CNTs are critical parameters to be controlled prior to their applications [97]. Since the discovery of CNTs, numerous physical and chemical techniques have been developed to attain these goals [98,99]. Polysaccharides with a broad range of characteristics, large-scale production, and low prices have shown to be highly suitable for CNT functionalization. The use of chitosan for CNT purification and functionalization has been proven to be a strategy to make drug release easier and more effective. Dou et al. [100] described a one-pot tactic for the development of chitosan-coated CNTs via a combination of Diels–Alder reaction and mercaptoacetic acid locking imine (MALI) reaction (Figure 11). Taking into account the broad use of Diels–Alder chemistry and MALI reaction, several carbon nanomaterials with different functional groups might be synthesized and applied to biomedicine.

Graphene and graphene oxide (GO) are 2D carbon-based nanostructures, in the form of nanosheets, that show an optimal combination of biocompatibility, strength, flexibility, and optical transparency, which made them suitable for the design of selective and sensitive sensors of biomolecules, which is crucial for medical sciences and the healthcare industry in order to assess physiological and metabolic parameters [101]. Besides, they show antibacterial and antiviral properties [96,102,103]. Graphene-based systems have proven to be effective via direct interaction with viruses and through photo-induced mechanisms, as well as platforms for other particles or molecules with antiviral properties. GO inactivates the virus by physical disruption: it can adhere to the structure of virus spikes and destroy them with the sharp edges of the GO layers. Its antiviral activity is effective on both DNA and RNA viruses, and depends on the concentration and incubation time. Reduced graphene oxide (rGO) and GO show similar antiviral activity, pointing towards a minor influence of the surface functional groups. The physical interaction of the viruses with their sharp edges seems to be the leading cause for the antiviral activity. Besides, they are negatively charged, which enables electrostatic interaction with the positively charged viruses. The higher interactions result in the destruction and inactivation of the virus. The viruses captured by GO have shown a loss of structural integrity: an RNA is released. The virus can then be identified using the recovered RNA [104]. Another method to inhibit the virus activity is using the GO photocatalytic activity. This approach has been developed by Hu et al. [105] to synthesize GO-aptamer nanosheets that were used to capture MS2 bacteriophage viruses, a small icosahedral nonenveloped RNA virus, which infects E. coli bacteria. This was used as a model for testing the antiviral properties of GO upon illumination with UV light. In this case, the leakage of the virus protein capsid predominates over the physical disruption produced by the sharp edges of the GO sheets.

**Figure 12:**
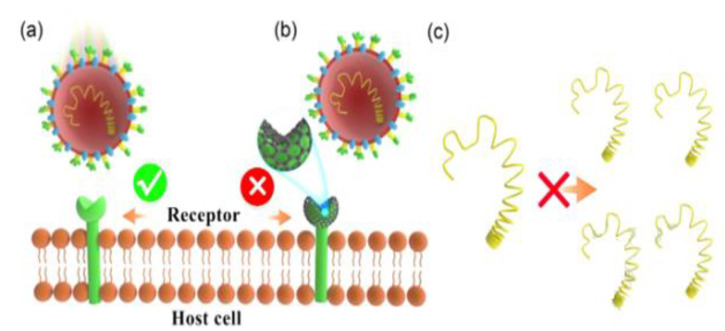
Scheme of the antiviral action of functionalized graphene quantum dots (GQDs). (**a**) Viral illnesses are caused by binding between the coronavirus (HCoV-229E) S-protein and the host cell receptor. (**b**) The presence of GQDs can prevent such binding. (**c**) This mechanism can inhibit the viral genome replication. Adapted from Ref. [107], copyright 2021, with permission from Elsevier.

Carbon quantum dots (CQDs), 0D carbon-based nanomaterials with fluorescence characteristics, also exhibit antimicrobial and antiviral properties [106]. These include amorphous carbon nanoparticles, graphene quantum dots (GQDs), partially graphitized core–shell carbon NPs, and amorphous fluorescent polymeric NPs. Their activity is attributed to the functional groups on their surface. CQDs functionalized with boronic acid demonstrated antiviral efficacy against HCoV-229E Human Coronavirus. HCoV-229E is an enveloped, single-stranded RNA coronavirus. It is one of the viruses that produce the common cold (Coronaviridae family, Human coronavirus 229E species), with a diameter in the range of 120–160 nm. Figure 12 shows two pathways for antiviral activity: (1) the attachment of CQDs (with a mean diameter of about 7 nm to the S-protein of viruses) to prevent infectious contacts between host cells and viruses; and (2) the capacity of CQDs to disrupt RNA genomic replication. Boronic acid functions were crucial in determining antiviral efficacy [107].

**Figure 13:**
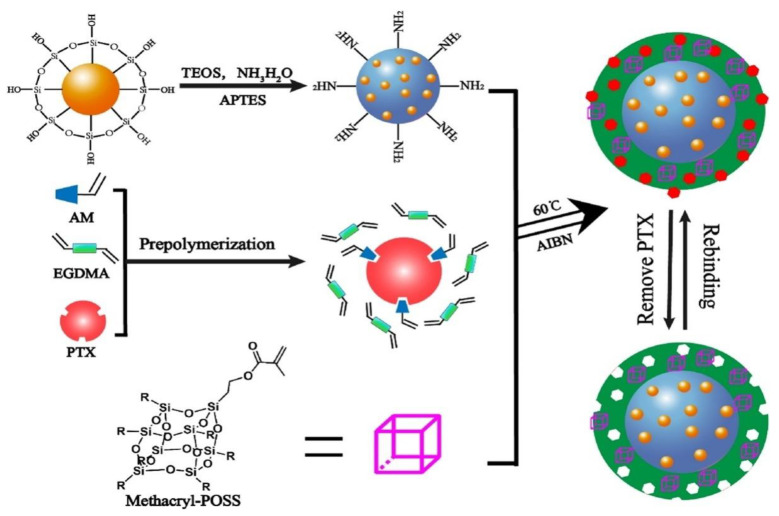
Schematic illustration for the preparation process and possible detection principle of the POSS-MIP/QDs. Adapted from Ref. [108], copyright 2021, with permission from Elsevier.

Bai et al. [108] developed a molecularly imprinted fluorescent sensor for selective identification of a model drug: paclitaxel. A molecularly imprinted polymer (MIP) shell was grafted on the surface of silane-functionalized Mn:ZnS QDs using a free radical polymerization procedure (Figure 13). Methacryl polyhedral oligomeric silsesquioxane (M-POSS) was utilized to provide a porous structure. Figure 13 depicts the synthesis process and the potential detection mechanism of the drug.

Van Tam et al. [106] used microwave-assisted pyrolysis of fructose to synthesize aniline-functionalized graphene quantum dots (a-GQDs). Then, phenyl boric acid (PBA) was used to modify the a-GQDs, leading to a fluorescence-quenching effect. The a-GQDs/PBA nanomaterial was tested as a fluorescence turn-on sensor for glucose detection, based on the specific interaction between PBA and glucose.

QDs also have great potential for cancer treatment. The selective attachment of FR-positive tumor cells with folic acid/folate (FA) was reported as a fast and easy technique for determining folate receptor (FR) expression in cancer cells. MKN 45, HT 29, and MCF 7 cancer cells were selectively marked using graphene quantum dots with folate coating and nitrogen doping (N-GQDs) [109]. DNA-functionalized QDs have drawn considerable attention in sensing and imaging, as well as cancer therapy [110]. Covalent conjugation, electrostatic interaction, direct dative interactions, and other ways for conjugating DNA to QDs have been documented in the literature [111]. In vitro photothermal imaging was described by Wang et al. [112] as AuNPs-QD complexes combined with DNA as a template. Horo et al. [52] developed DOX-loaded chitosan-AuNPs and beads, both of which were implanted with functionalized silk fibroin. Chitosan was used as a reduction and stabilizing agent to synthesize NPs with dimensions in the range of 3-8 nm. Compared to uncoated materials, coated materials demonstrated a delay in drug release. As a result, drug delivery strategies based on functionalized silk-coated substances may be useful for producing localized and protracted drug release.

### 3.5. Polymeric Nanomaterials

Polymeric NPs are colloidal particles in the range of 10 nm–1 μm made up of polymers that can be straightforwardly synthesized through chemical reactions in order to tailor the loading and release of drugs and genes. The benefits of these NPs are their easiness to synthesize, high stability, biodegradability, nontoxicity, lengthy blood circulation time, and sustained and targeted delivery. Furthermore, they can be tailored according to their shape, size, surface functional groups, degree of porosity, as well as their mechanical characteristics [113]. They are divided into three main groups: natural, biosynthesized, and chemically synthesized. They can be fabricated into different shapes, including liposomes, dendrimers, nanospheres, nanocapsules, nanogels, and micelles (Figure 2). They are used in wound dressings, pharmaceutical excipients, medical devices, dental materials, and scaffolds [114]. Biodegradable polymers frequently used for the development of polymeric NPs are poly(lactide) (PLA), poly(ɛ-caprolactone) (PCL), PLGA and polycarbohydrates such as alginate, chitosan, and gelatin.

Overall, because of their excellent chemical, physical, and mechanical properties and their versatility of synthesis, functionalized nanomaterials can be employed in a variety of ways. Although functionalized nanoparticles are hardly used in the industrial field up to date, they can aid in developing novel concepts in a variety of industries. Functionalized nanomaterials promise to produce better and cost-effective consumer products and industrial operations. An inappropriate use can have a detrimental effect on surroundings, public health, and safety in various ways [115,116,117].

## 4. Cytotoxicity: The Role of Functionalization

**Figure 14:**
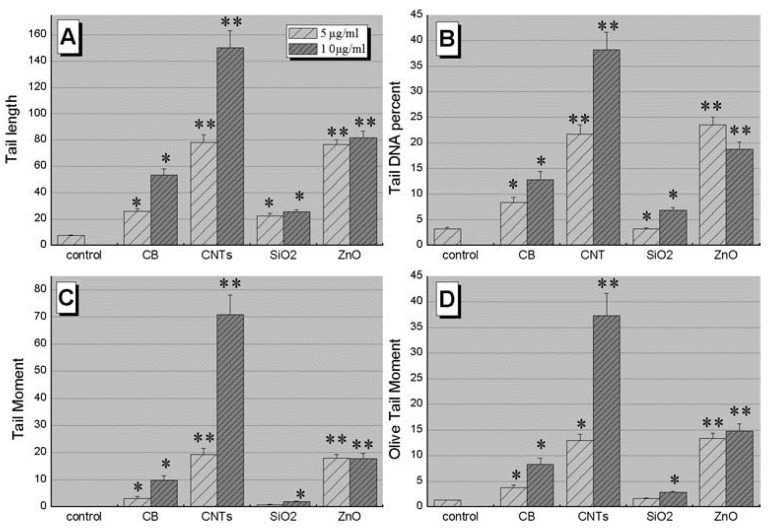
DNA damage determined by comet assay in PMEF cells exposed to NPs. Cells were respectively treated with 5 μg mL^−1^ of CB, CNT, SiO_2_, and ZnO for one day. Damage was evaluated by (**A**) tail length, (**B**) tail DNA, (**C**) tail moment, (**D**) Olive tail moment. Values shown are the mean from 50 images. * *p* < 0.05; ** *p* < 0.01 in comparison to blank. Adapted from Ref. [121], copyright 2009, with permission from Wiley & Sons, Inc.

Chemical composition, crystalline structure, size, and density are parameters that strongly influence nanomaterial toxicity and cytotoxicity [28,118]. Nanomaterial absorption and intracellular localization can be linked to some health hazards due to the nature of nanomaterials and their chemical interactions with cells. Chemical composition, for example, might cause oxidative stress in cells [119]. CNTs are believed to be more poisonous than carbon black or silica nanoparticles and can induce severe lung damage [120]. Asbestos is less hazardous than TiO_2_, Fe_3_O_4_, and ZrO [121]. Another indicator of cytotoxicity based on membrane integrity damage is lactate dehydrogenase (LDH) leakage. Additionally, DNA damage in primary mouse embryofibroblasts (PMEF) treated in vitro with different amounts (5, 10, 20, 50, and 100 μg mL^−1^) of manufactured nanoparticles (Figure 14) revealed that CNTs and ZnO caused more DNA damage than carbon black (CB) and SiO_2_ NPs [121]. 

The crystalline structure also has a strong effect on NPs’ toxicity [122]. For instance, TiO_2_ NPs, which can naturally appear in three different crystalline forms, i.e., anatase, rutile, and brookite, are reported to have cytotoxic and genotoxic effects. Rutile titania is slightly more lethal than anatase TiO_2_ NPs. This might be elucidated considering the different reactivity of the two forms: rutile TiO_2_ NPs are more photocatalytic than anatase and therefore, are capable of producing larger quantities of oxygenated free radicals on their surface. On the other hand, other allotropes have a significant influence on cell viability and, as a result, on human health. Sato et al. [123] discovered that TiO_2_ allotrope toxicity is affected by the NPs’ environment’s ambient conditions. In the absence of UV radiation, rutile TiO_2_ NPs (200 nm) caused oxidative DNA injury, whereas TiO_2_ NPs (10–20 nm) caused oxygen-reactive species (ROS) generation.

**Figure 15:**
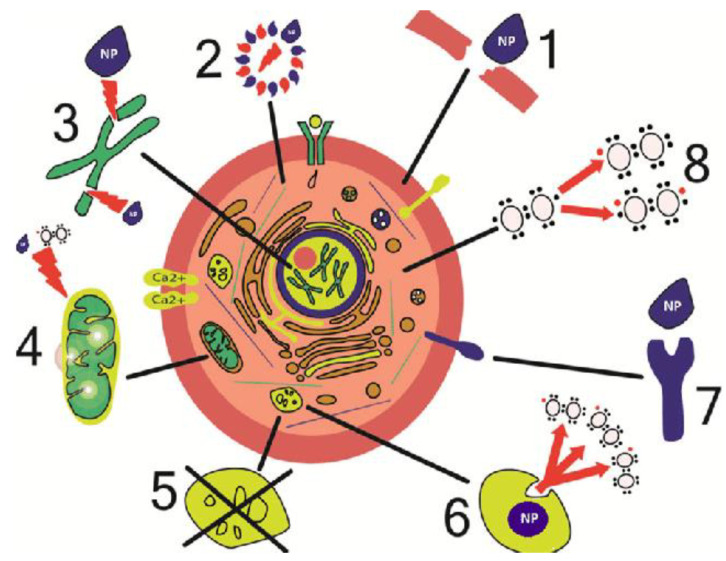
Mechanisms of cell damage by nanoparticles. (1) Physical damage of membranes. (2) Structural changes in cytoskeleton components. (3) Disturbance of transcription and oxidative damage of DNA. (4) Damage of mitochondria. (5) Disturbance of lysosome functioning. (6) Generation of reactive oxygen species. (7) Disturbance of membrane protein functions. (8) Synthesis of inflammatory factors and mediators. Adapted from Ref. [124], copyright 2018, with permission from Springer Nature.

Another key component in minimizing nanomaterial toxicity is particle size [124]. Nanoparticles with a smaller size are more prone to pass through biological barriers. Phagocytosis or other pathways can facilitate the entrance of small NPs to cells. NPs can discriminate between adhesive connections because of their ability to infiltrate cells. It can produce forces such as Van der Waals, steric interactions, and electrostatic charges [125]. Furthermore, unlike big nanoparticles, NPs in the size range of 1 to 100 nm are not phagocytized but instead taken up via RME routes. In the lack of specific cell surface receptors, NPs can be absorbed. Most cells can effectively assimilate NPs with size of 50 nm or smaller (causing cytotoxicity). NPs smaller than 20 nm can easily pass through blood arteries and concentrate in organs [126]. NPs with a large surface area, such as NiO (diameter < 25 nm), clump together in liquids, and engage and induce oxidation and DNA damage by interacting with molecules including proteins and DNA [127]. The mechanisms of cell damage by NPs are depicted in Figure 15 [124]. 

## 5. Cost-Effective Functionalization

**Figure 16:**
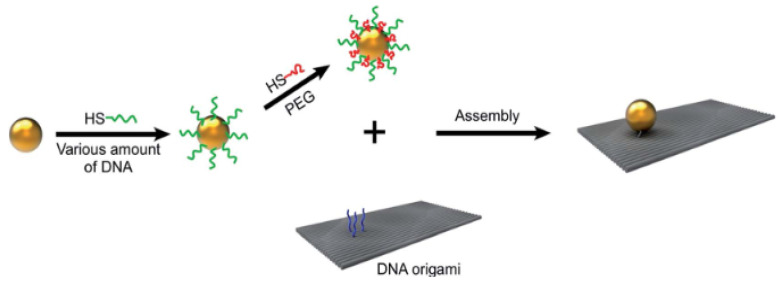
AuNPs are functionalized in two stages: first with DNA/PEG polymers comprising variable amounts of DNA, and then with rectangular DNA origami. Adapted from Ref. [130], copyright 2017, with permission from The Royal Society of Chemistry.

The functionalization of AuNPs using a mixture of DNA and PEG polymers is the most cost-effective and satisfactory method available for nanomaterial cofunctionalization [128,129]. To obtain a comparable level of gold NP binding effectiveness with DNA origami nanostructures, Wang et al. [130] used a significantly smaller amount of thiol-DNA in their technique than pure DNA functionalization. Because of the decreased DNA consumption and lower costs, the use of DNA–NP conjugates in nanotechnology can be scaled up. Figure 16 shows the functionalization process of AuNPs with DNA/PEG polymers [130].

## 6. Applications of Functionalized Nanomaterials in Biomedicine

**Figure 17:**
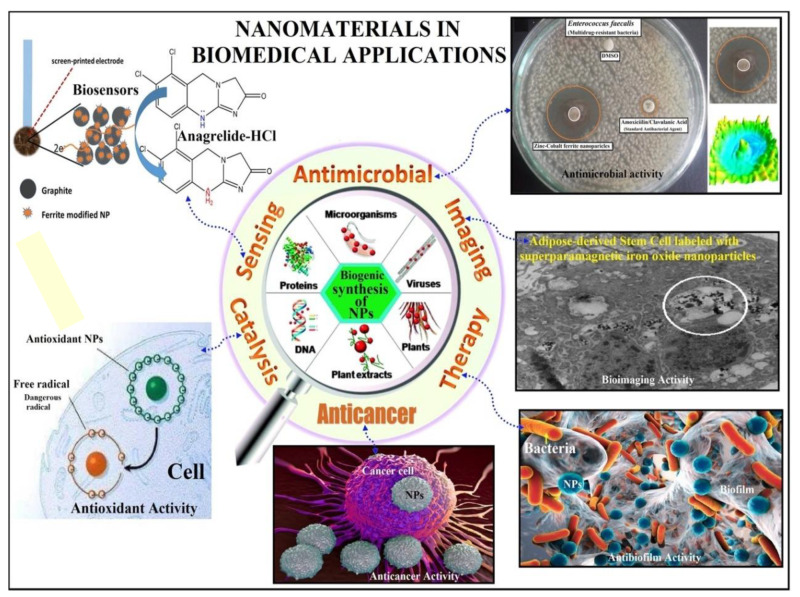
Summary of the applications of nanomaterials in biomedicine. Adapted from Ref. [135], copyright 2019, with permission from Elsevier.

Medical diagnosis [131], immunization [132], treatment [133], and even healthcare services have been transformed and influenced by nanotechnology [134]. Chemical functionalization, physical functionalization, and surface synthesis link biological agents with various NPs. It is possible to classify the biomedical applications of nanotechnology into different areas, as summarized in Figure 17 [135]. Additionally, some relevant examples have been provided for each category in Table 1.

### 6.1. Diagnostic Implications of Functionalized Nanomaterials

**Figure 18:**
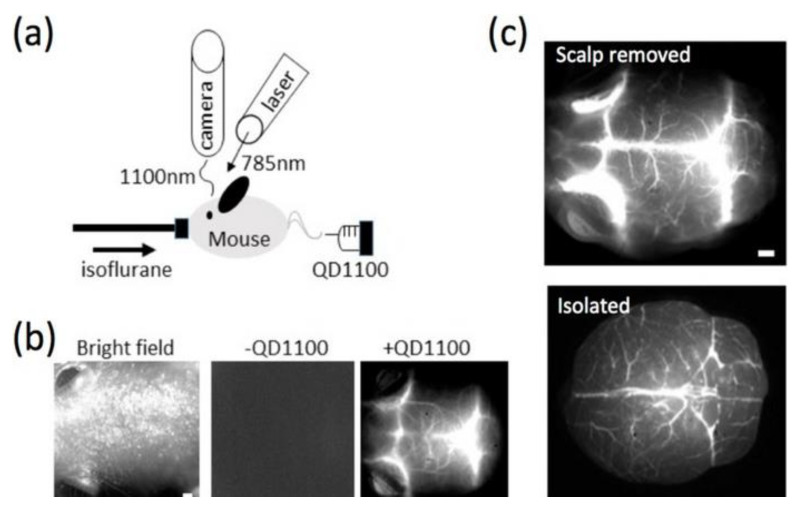
(**a**) Setup for NIR fluorescence imaging of cerebral arteries. (**b**) Imaging of a mouse head. Bright field micrograph (**left**), NIR fluorescence image without (**middle**), and with QDs (**right**). (**c**) NIR fluorescence pictures of cerebral blood vessels. The upper image shows the fluorescence after the scalp has been removed, whereas the lower micrograph shows the fluorescence after separation—with one-millimeter scale bars. Taken from Ref. [152], copyright 2016, with permission from MDPI.

Nanomaterials are extensively employed in imaging modes, such as optical coherence tomography and MRI. QDs are semiconductor nanocrystals commonly employed in optical imaging [151]. Imamura et al. [152] used PbS QDs for noninvasive scanning of septic encephalopathy in mice, suggesting that these nanomaterials can be used to image a variety of vascular systems. NIR fluorescence imaging of the mouse brain during therapy with Pbs QDs is shown in Figure 18 [152]. Before administration of QDs, only low-intensity NIR fluorescence signals were distinguished (Figure 18b, middle), due to the extremely low background fluorescence in this spectral zone. When QDs were intravascularly inserted into the mouse, the fluorescence signals arising from the mouse head augmented, and the vascular structure of cerebral blood vessels became visible (Figure 18b, right) 

The development of nanoparticles with fluorescence characteristics for in vivo imaging is currently in progress. Because silicon nanocrystals are cell-safe, abundant, and more appealing than QDs [153], they do not necessitate a dense surface coating to protect the nanocrystal center from oxidation and the environment.

### 6.2. Therapeutic Applications of Functionalized Nanomaterials

**Figure 19:**
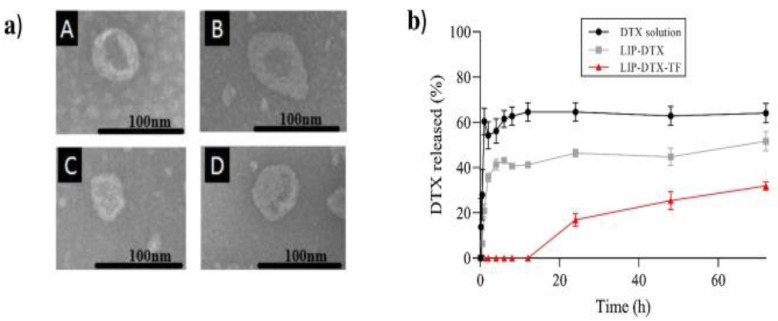
(**a**) Transmission Electron Microscopy of liposomes: (**A**) empty liposomes (LIP); (**B**) docetaxel-loaded liposomes (LIP-DTX); (**C**) empty transferrin functionalized liposomes (LIP-TF); and (**D**) docetaxel-loaded liposomes functionalized with transferrin (LIP-DTX-TF). (**b**) In vitro release profile of free DTX and encapsulated in liposomes in PBS buffer pH 7.4. Adapted from Ref. [30], copyright 2021, with permission from Elsevier.

Magnetic nanoparticles, AuNPs, and CNTs have been utilized in the field of biomedicine. The application of NPs in postoperative treatment has attracted the attention of many researchers [153]. The use of superparamagnetic Fe_3_O_4_, GO, and doxorubicin-incorporated nanofibers has been claimed to reduce the localized regression of breast cancer and develop tissue regeneration [60]. A functionalized peptide that provides specific drug delivery possibilities with improved drug permeability, noteworthy aggregation in the desired target, and high therapeutic efficacy can help with the liposomal formulation in cancer treatment [20]. Docetaxel is a widely used anticancer chemotherapy drug, and transferrin is a blood–plasma glycoprotein that plays a key role in iron metabolism. Fernandes et al. [30] synthesized docetaxel-loaded liposomes functionalized with transferrin (LIP-DTX-TF), and their effects on prostate neoplasms were studied. TEM images demonstrated that the systems were spherical and nanometric in size (Figure 19a) and that the presence of DTX aided in vesicle size reduction, resulting in improved liposome stability (Figure 19b).

## 7. Functionalized Nanomaterials: Drug/Gene Delivery

**Figure 20:**
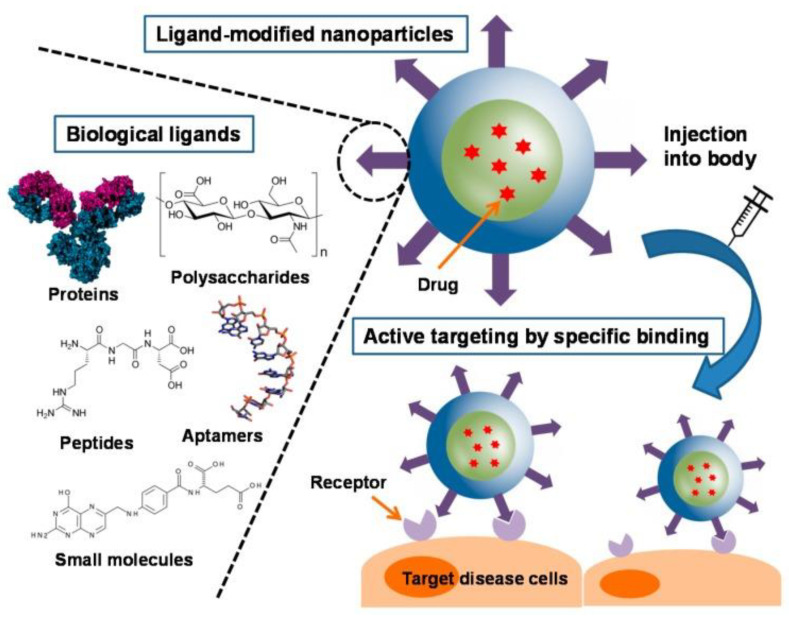
Illustration of biological ligands for active targeting of nanoparticle drug carriers. Taken from Ref. [161], copyright 2019, with permission from MDPI.

Nanomaterials can be functionalized for different purposes, including drug delivery carriers or therapeutic agents for cure and treatment, diagnostic applications in biological imaging, cell labeling, biosensors, and the use of moieties for medical devices such as stents or lenses [154,155,156]. Functionalization can improve biocompatibility and uptake efficiency and simultaneously minimize immune system activation, increasing the material’s bioavailability inside the body. These modifications are beneficial for some drug delivery strategies to ensure that the appropriate doses of the drug are released to the correct area while limiting the detrimental effects of drug molecules on other organs [157]. Drug delivery systems are necessary to improve the efficacy of drug biodistribution. Nanomaterials have been used to carry drugs and genes in passive, active, and direct methods [49,158,159]. Due to the small size of nanoparticles, they can pass across cellular membranes and boundaries. Moreover, the increased surface-to-volume ratio of nanoparticles leads to improved drug loading [160]. Figure 20 displays biological ligands used for active targeting of NP drug carriers [161], and Table 2 summarizes different functionalized nanomaterials applied in drug/gene delivery.

## 8. Functionalized Nanomaterials: Regenerative Medicine

Reparative and restorative medicine and nanotechnology have gained popularity in recent years, resulting in significant improvements in medical research and clinical practice [180]. Tissue engineering, cell therapy, diagnostics, medication, and gene delivery are examples of regenerative medicine applications that use various functionalized nanoscale materials [24,181,182]. Restorative medicine is a vast field of nanotechnology that strives to regenerate cells and tissues similar enough to their original design and function. Three main types of therapeutic techniques can be found in regenerative medicine: tissue-engineering treatments based on cells; biomaterials; and a combination of the two. Stem cell biology, nanotechnology, and bioengineering have progressed significantly, potentially paving the way for real regenerative medicine for various diseases [183]. Stem cells are known for their capacity to maintain their differentiating potential while intersecting to generate numerous daughter cells. Such daughter cells lack “stem-ness” and use controlled proliferation to produce adult cells of all origins throughout the body (self-renewal) [184]. Using tissue-specific or therapeutic genes, as well as primary cells that overexpress these genes, genetically engineered cell treatment can manufacture proteins with a therapeutic intent, to be used at regeneration platforms or discriminate new cells into the appropriate cellular lineage, assisting in tissue restoration [185].

**Figure 21:**
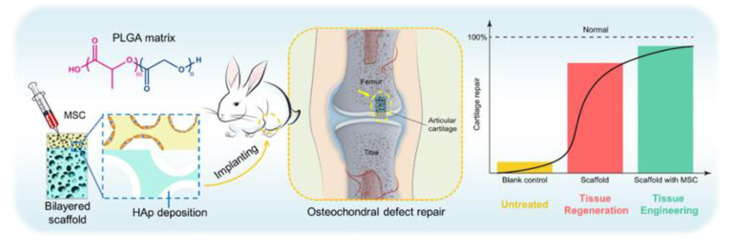
Illustration of hydroxyapatite-based scaffold-induced regeneration of bone. Reprinted from Ref. [189], copyright 2018, with permission from the American Chemical Society.

When bone is formed, it comprises mostly collagen fibers and calcium phosphate, which is converted into hydroxyapatite (HDA). Bone tissue also contains several cellular structures, such as osteoblasts, osteocytes, and osteoclasts, which contribute to its calcification [186]. For bone repair, nanoscaffolds with adequate biophysical characteristics, such as stiffness and cell proliferation, have been employed. A variety of nanofiber matrices have been synthesized in recent years. The vast majority of nanoscaffolds are developed to match genuine bone’s structural, compositional, and biological features [187]. Zhang et al. [188] prepared a chitosan/HDA biomimetic nanocomposite scaffold for assessing the effect of bone marrow MSC mesenchymal stem cells (BMSCs) growth, and explored the molecular mechanism both in vivo and in vitro. It was reported that this hybrid scaffold could encourage the proliferation of BMSCs and trigger the integrin-BMP/Smad signal pathway of BMSCs. In addition, HAD can also be used combined with other polymeric materials such as PEG, PCL, and PLGA, which have displayed improved effects in bone regeneration/repair (Figure 21) [189,190].

Besides nano-HDA, collagen, electrospun silk, anodized titanium, and nanostructured titanium surfaces are some of the primary constituents of materials that mimic the bone extracellular matrix [191]. In primary osteoblasts, nanofibers have improved osteogenesis and biomineralization. Main osteoblasts are limited in their application due to (i) restricted accessibility and intrinsic donor site malady; (ii) limited scaling capability; (iii) age-related behavior; or (iv) possibility of dedifferentiation occurring during in vitro cultivation [94].

Transient gene delivery [192], cell therapy without the need for genetic modification [193], and genetically modified cells [194] are currently three of the most exciting new procedures in the field of tissue engineering. Gene delivery is a therapeutic approach to introducing foreign genetic material directly into host cells in vivo. These genes immediately affect the host tissue, causing it to remodel [195].

Achieving better cell adhesion, motility, and differentiation through nanomedicine is possible thanks to the development of interfaces, components, and substances that mimic the cells’ natural environment. Scientists have developed complex tissue/organ constructions by combining stem cells with scaffolds and stimulating factors as the basis of their tissue-engineering experiments [145,196]. Some of these are currently being utilized therapeutically as part of the standard treatment for various disorders. Scaffolds are transformed into three-dimensional structures that have the appropriate shape, size, architecture, and physical properties for different applications and environments. For this reason, tissue-engineering products are designed to look and behave like natural tissues. In addition to biocompatibility and controllable porosity and permeability, important scaffold characteristics include mechanical and degradation kinetics comparable to those of the desired tissue and support for cell adhesion and proliferation by adding nanotopographies to the biomaterial surface [197].

Biodegradability is a critical property that nanoparticles must possess to be employed safely inside the body. This is a crucial aspect to consider when building scaffolds for tissue engineering and reparative and restorative medicine [198]. Table 3 summarizes functionalized nanomaterials that have been utilized in tissue engineering.

## 9. Functionalized Nanomaterials: Cancer Therapy

**Figure 22:**
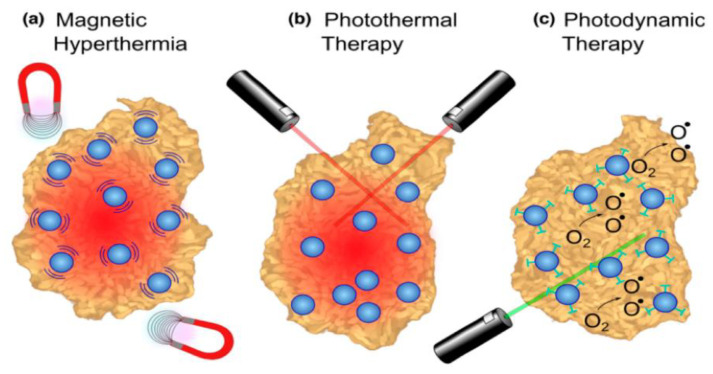
Schematic representation of tumor ablation therapies with iron oxide nanoparticles (NPs). Reproduced from Ref. [215], copyright 2016, with permission from Elsevier.

Theranostic nanoprobes for tumors and malignancies have become a prominent focus of research since NP functionalization has been able to be used simultaneously in diagnostic and therapeutic purposes. Surface modification of NPs has been proven to generate targeted accumulation in tumor tissue due to the enhanced permeability and retention (EPR) effect [29,213]. Tumors have more permeable vasculature, a poorly defined lymphatic system, and various substances that aid in increased targeting, as contrasted to normal tissue, such as VEGF and basic fibroblast growth factor [214]. In cancer immunotherapies, NPs can keep track of critical immune cells during metastasis. Different tumor ablation therapies with magnetic NPs such as Fe_3_O_4_ have been reported [215] (Figure 22): (a) Magnetic hyperthermia, in which an alternating magnetic field induces NPs to produce heat, boosting tumor necrosis. (b) Photothermal ablation, in which the light absorbed by the NPs is transformed into thermal energy, producing cell death in the neighborhood. (c) Photodynamic therapy, in which photosensitizing agents anchored to NPs are activated via an external light source to make singlet oxygen species that are cytotoxic to cells. As a result, NPs have a high level of target-cell selectivity [216]. Table 4 displays functionalized nanomaterials that have been utilized for cancer treatment.

## 10. Functionalized Nanomaterials: Medical Implants

**Figure 23:**
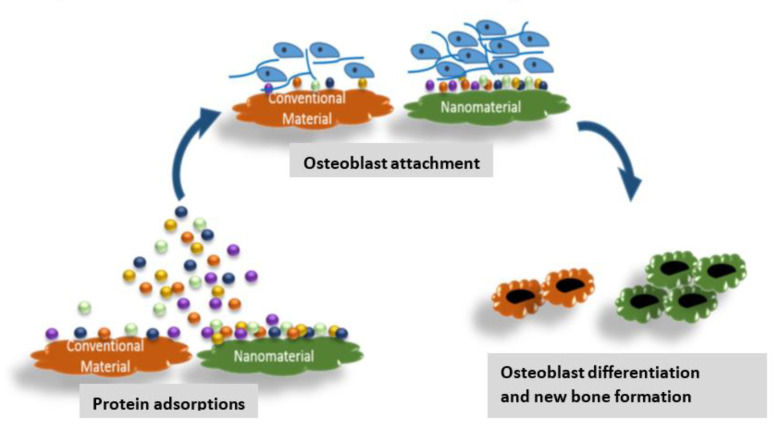
Comparison of bone regeneration using nanomaterials and traditional materials. Nanomaterials show improved protein adsorption, osteoblast anchoring, and differentiation compared to traditional materials. Reproduced from Ref. [142], copyright 2020, with permission from Elsevier.

Recently, the influence of nanotechnology on the implant field has increased strongly. Nanomaterials with biological-inspired structures are motivating scientists to investigate their potential for enhancing the performance of conventional implants [91,142]. Nanotechnology has the skill to economically substitute many traditional implants and offer numerous novel applications. It can result in more efficient and longer-lasting implants, with reduced infection rates and enhanced bone or tendon healing. In orthopedics, the goal of biomaterials is to substitute injured bone. Improved mechanical properties (e.g., strength, flexibility, hardness, elastic modulus), wear, hydrolysis and corrosion resistance, biocompatibility, osseointegration, bioinertness, and ease of surgical application are required properties to be used in orthopedics [226,227]. Nanomaterials offer an enlarged surface area, a superior stiffness, and a high roughness that can improve the adhesion and proliferation of bone-related proteins and the deposition minerals incorporating Ca [228]. Besides, FMNs can mimic the amounts of the components of natural bones and can aid in sustaining biologically active growth factors and exploit the potential of BMSCs. Numerous studies [229] have been developed to examine the optimal surface properties of FMNs that may support or assist specific protein adsorption, improved osteoblast anchoring, osteoblast differentiation, and new bone formation (Figure 23) [142].

Surface adjustment of nanomaterials is a prospective method to expand the performance and durability as well as to reduce the hazardous side effects that might take place during implant degradation. Surface characteristics have a key role on modulating biological interactions. Specifically, engineered nanomaterials can have a significant impact on molecular and cellular actions; this issue aids in conditioning the comprehensive biological response of an implant (i.e., protein adsorption, cell adhesion, and proliferation). Therefore, several approaches have been settled to modify nanomaterials for orthopedic implants such as anodic oxidation [230], plasma electrolytic oxidation [231], electrochemical plating [232], chemical conversion coating [233], physical vapor deposition, laser surface alloying [234], thermal spraying [235], organic coating [236], and so forth. These methods provide new implant surfaces with tailorable characteristics at the nanoscale. The particular procedure can be chosen based on different factors/goals, including to attain complex geometries and to be suitable for large-scale processing. Metal oxide NPs such as TiO_2_, ZrO_2_, and Al_2_O_3_ have been used as nanocoatings to enhance the mechanical and biochemical properties of conventional metallic implants [237].

## 11. Conclusions

Nanotechnology has opened up vast techniques to manipulate and transform the current medical devices or materials utilized for therapy in biomedical sciences and engineering. Numerous nanomaterials can be used in biomedical applications, both organic, such as CNTs, GO, GQDs, and polymeric NPs, and inorganic, such as metallic NPs (Au, Ag), metal oxide NPs (TiO_2_, Fe_3_O_4_, mesoporous SiO_2_), and ceramic (HAD, CaCO_3_). Over the last years, numerous approaches have been developed to synthesize surface-engineered nanomaterials, in particular NPs, for drug/gene delivery, diagnostics, cancer therapy, tissue engineering, and medical implants, and the structure–function relationship of these functionalized nanoparticles has been widely explored. The NPs’ surface modification is a potent strategy to improve biocompatibility and uptake, as corroborated by the huge quantity of scientific documents published on this subject. Investigations prove that the conjugation of polymers, biomolecules, and small ligands on the NP surface can successfully increase biocompatibility both in vivo and in vitro, due to the alteration of surface charge and to the inactivation of sensitive functional groups that can influence the stability of the cell membrane. Besides, the incorporation of certain molecules can improve NPs’ passive and active uptake, reducing systemic toxicity in vivo and enabling high precision therapy and/or diagnosis. The binding of functionalization agents on the NP surface can be achieved via covalent and noncovalent tactics. The first is broadly used to link proteins, antibodies, aptamers, and peptides utilized to boost uptake and to achieve active targeting, whereas the second is frequently used for the loading of drugs and other molecules that need to be liberated into the cells. The promise of tissue and organ-specific regeneration therapy has become a reality due to major advances in regenerative medicine and nanomedicine over the previous decade. Preliminary clinical results have shown that functionalization of NPs with specific recognition surface moieties results in improved efficacy and reduced side effects, due to properties such as directed localization in tumors and active cellular uptake. Even though remarkable improvements have been attained, this research arena is still in its early stages, and significant efforts are needed in order to be able to scale up the functionalization approaches developed at the laboratory level and make them reproducible. A prerequisite for progressing in this research area is the development of novel chemical methods to conjugate chemical moieties onto NPs in a safe and consistent manner. In addition, smart and innovative nano-based technologies can offer particular physicochemical properties that could aid in fixing crucial issues associated with the treatments of viral infections such as SARS-CoV-2. Researchers may find this study valuable in analyzing past studies on the topic matter to attain commercial success. 

## Figures and Tables

**Table 1:** Applications of functionalized nanomaterials in nanomedicine.

Application	Example	Ref.
Diagnostic Imaging	X Ray Tomography Magnetic resonance imaging Photothermal imaging	[136] [137] [138] [112]
Therapy	Drug delivery Gene and stem cell therapy Hair growth	[139] [140] [141]
Medical implants	Orthopaedic Cardiovascular Neurological Dental	[142] [143] [144] [145]
Tissue Engineering	Bone Cartilage	[54] [146]
Anticancer	Paclitaxel DOX Docetaxel Gambogic acid	[108] [60] [30] [147]
Sensing	Glucose Insulin Metabolic biomarkers	[106] [148] [38]
Antimicrobial and Antiviral	Streptomycin, penicillin Coronavirus *E. coli* Airborne viruses	[149] [107] [104] [150]

**Table 2:** Functionalized nanomaterials used for drug/gene delivery.

Nanomaterial	Function	Size (nm)	Drug/Gene	Target Organ & Indication	Ref.
Porous CaCO_3_	Intranasal drug carrier	2000–3200	Insulin	Postprandial hyperglycemia in diabetes	[162]
CaCO_3_ NPs	Drug/gene delivery	116	Ciprofloxacin HCl	S. Aureus	[163]
CaCO_3_	Drug delivery	40–200	Hydrophilic drugs and bioactive proteins (validamycin)	Inflamed region	[164]
Cationic NPs	Gene delivery	50–100	Raf gene, ATP^μ-^Raf	Angiogenic blood vessels (tumor-bearing mice)	[165]
Fe_3_O_4_@GO	Drug release and antitumor therapy	200–1000	Hybrid microcapsule	Tumor cells targeting	[166]
GO flakes	Drug release	1000–2000	DOX microcapsules	-	[167]
AuNPs	Drug delivery	100	--	Nasopharyngeal carcinoma cells	[168]
FA-Au-FITC ^1^	Drug delivery for cancer therapy	4–7	DOX	Cytoplasm	[169]
HLA ^2^-Si/Fe_3_O_4_ NPs	Drug delivery for cancer therapy	40–110	DOX	Tumor tissues	[170]
Fe_3_O_4_-SA-PVA-BSA ^3^	Drug delivery	240–460	DOX	Cancer cells	[171]
CS-HYL-5-FU-PEG-G ^4^	Drug delivery	300–580	COLO-205 and HT-29 colon	Cancer cells	[172]
SA/PVA/Ca ^5^	Drug delivery system	500–1000	Diclofenac sodium	-	[173]
PLGA ^6^-Fe_3_O_4_	Drug delivery system	67	5-Fluorouracil	Prostate carcinoma cell	[113]
HLA-Nanoemulsion	Drug delivery system	–	Ciprofloxacin	-	[174]
Fe_3_O_4_	Drug delivery system	20	Gambogic acid	Capan-1 pancreatic cancer cells	[147]
PLGA-Fe_3_O_4_ NPs	Intratumoral drug delivery	200–300	DOX	Murine Lewis lung carcinoma cells	[175]
Fe_3_O_4_ conjugate oleate/oleylamine	Drug release	12	Chromone	HeLa cells	[176]
Fe_3_O_4_/DPA-PEG-COOH ^7^	Drug delivery	9	Dextran, PEG	Macrophage Cells	[177]
Thiolated starch-coated Fe_3_O_4_	Drug delivery	40–50	Isoniazid	Human body cells	[178]
Zn-doped Fe_3_O_4_ nano-octahedral core	Drug delivery	10–20	DOX and HSP70/HSP90 siRNAs	Tumor cells	[138]
Arginine-NCQDs ^8^	Gene delivery	6–11	EGFP gene	Mammalian cells	[179]

^1^ Folic acid-coated gold nanoparticles conjugated with a fluorophore; ^2^ Hyaluronic acid-modified mesoporous silica-coated Fe_3_O_4_ NPs; ^3^ Fe_3_O_4_ nanoparticles coated with a mixture of sodium alginate (SA), polyvinyl alcohol (PVA), and bovine serum albumin (BSA); ^4^ Polyethylene glycol-gelatin-chitosan-hyaluronidase-5-fluorouracil; ^5^ Sodium alginate/polyethylene glycol (vinyl alcohol); ^6^ Poly(lactic-co-glycolic acid); ^7^ Dopamine-polyethylene glycol-carboxylic acid; ^8^ Nitrogen-doped carbon quantum dots.

**Table 3:** Functionalized nanomaterials utilized in tissue engineering.

Nanomaterial	Function	Size (nm)	Tissue	Purpose & Outcomes	Ref.
PEG-GO	Tissue engineering	50	Bone	Improved thermal stability, hydrophilicity, water absorption, biodegradation, mechanical, viscoelastic, and antibacterial properties	[54]
Oxidized alginate/ gelatin hydrogel	Tissue regeneration	100–200	Cartilage regeneration for the treatment of osteoarthritis	Usefulness of the hydrogel in encouraging cellular migration and proliferation	[146]
OCMC ^1^	Tissue engineering	2000–4000	BALB/c3T3 cells in rates	Biocompatibility, spinnability of hydrogel through electrospinning	[199]
Pd/PPy/rGO NC ^2^	Tissue engineering	2–4	Bone	Biocompatibility, osteoproliferation, and bacterial infection prevention	[200]
3D macro-rGO/PPY	Bone tissue engineering	100–400	Backbone	Casein phosphopeptide as bioactive for bone engineering, osteoblastic performance, biological properties	[201]
Chitosan-ZnO	Soft tissue engineering	180		Improved hydrophilicity, porosity, water absorption, oxygen permeability, biodegradability, antibacterial and wound healing	[202]
Biphasic Calcium Phosphate	Bone tissue engineering	1–2	MG63 cells	Micropores and collagen coating influence cellular function, in vitro cellular behavior, scaffold–osteoblast interactions	[203]
AuNPs/glass-ceramic matrix	Bone tissue engineering	5–10	Bone	In vitro hydroxyapatite synthesis, controlled release of gold species, biocompatibility, and antibacterial activity of AuNPs	[91]
AuNPs	Tissue engineering	20	Rat brain	AuNP biochemical effects on the rat brain, biomarkers of AuNP toxicity	[204]
AuNPs	Tissue Engineering	10–50	Cardiac tissue	Effects of AuNPs on the histological deformities of rat heart tissue, toxicity, therapeutic and diagnostic potential of NPs, and their interaction with proteins and other cells	[205]
AuNPs	Tissue engineering	30 nm	Subsets of cells in human organs	NP toxicity in human blood, hemolysis, development of ROS ^3^, platelet condensation in cell subsets	[206]
AuNPs/polymeric coatings	Tissue engineering	18, 35, 65	Endothelial cells from human dermis	NP toxicity, uptake behavior, and uptake quantification	[207]
Bioactive glass scaffolds	Tissue engineering	50–100	Bone repair	Osteoblastic cells for bone reconstruction	[208]
Na_2_Ca_2_Si_3_O_9_	Bone tissue engineering	500	Bone	Bioactive and biodegradable scaffold effects, mechanical support	[209]
Bioactive glass- ceramics/apatite	Bone tissue engineering	8–20	Bone	Crystallization rate of bioactive glasses on the kinetics of HAD formation	[90]
Ca_10_(PO_4_)_6_(OH)_2_	Bone tissue engineering	1000–2000	Trabecular bone	Extent and nature of carbonate substitution on HDA	[93]
GO/Chitosan Scaffold	Cardiac tissue	––	Cardiac tissue	Investigate cell survival, cell adhesion, development of intercellular networks, genes, and proteins expression	[210]
GO/Chitosan Scaffold	Cartilage repair	35–60	Cartilage tissue	Nanocomposite effect on human tissue, effects of GO	[211]
GO-coated collagen scaffolds	Tissue engineering	––	Mouse osteoblastic MC3T3-E1 cells	Influence of the GO coating on cell growth and differentiation, biocompatibility and biodegradability of collagen scaffolds, bioactivity studies	[212]
Nanocrystalline apatite/AuNPs	Tissue engineering	2–25	Bone tissue reconstruction	Toxicity of NPS in simulated physiological fluid	[66]

^1^ Gelatin − oxidized carboxymethyl cellulose. ^2^ Nanocrystalline cellulose. ^3^ Reactive oxygen species.

**Table 4:** Functionalized nanomaterials used for cancer therapy.

Nanomaterial	Functionalization Agent	Size (nm)	Drug	Purpose & Outcomes	Ref.
ZnO NPs	PBA	40	Curcumin	High drug-loading and release rates, in vitro and in vivo antitumor efficacy	[88]
AuNPs	Beta-cyclodextrin with PEG, biotin, PTX, rhodamine B	30–50	PTX	Cytocompatibility, stability, and biomolecule binding ease	[217]
SPION	5TR1 Aptamer	57	Epirubicin	Magnetic resonance (MR) traceability, nontoxicity, increased permeability, retention effect	[82]
Fe_3_O_4_ NP_S_	Glycerol monooleate	144	PTX, rapamycin, alone or combined	Intravenous administration of hydrophobic drugs	[218]
rGO ^1^	Fe_3_O_4_ NPs	54.8	Camptothecin	pH-responsive drug release profile, good biocompatibility, excellent photodynamic	[219]
Fe_3_O_4_ MNPs+ PLGA	citric acid	130–140	DOX, verapamil	Loading hydrophilic and hydrophobic drugs	[220]
MSN ^2^	β-cyclodextrin with hydroxyl, amino, and thiol groups	75.5	DOX	Higher mucoadhesive on the urothelium	[221]
rGO	HA-PEG-g-poly(dimethylaminoethyl methacrylate)	120−190	-	Biocompatibility, in vitro cellular uptake sensitive to cancer cells	[222]
MSN	Galactose	277	Camptothecin	MSN targeting to cancer cells	[59]
rPEI- Cdots ^3^	FA	143	-	Biocompatible, good siRNA gene delivery carrier	[223]
PLGA NPs	bis(sulfosuccinimidyl) suberate (BS3)	184	Curcumin	Promote the loading of low-soluble drugs and aid in sustained released	[114]
ZnO NPs	PBA	414	Curcumin	Curcumin distribution to the sialic acid is much easier by PBA conjugation	[88]
Se NPs	(Arg–Gly–Asp–d-Phe–Cys [RGDfC]) cyclic peptide	18	DOX	Antitumor efficacy in vivo, effective cellular uptake A549	[224]
CuO NPs	FA, starch	108.83	Cytochrome C	Antioxidants, anticancer, antimicrobial, drug-carrier	[35]
MoS_2_	FA, BSA	133	DOX	Excellent photothermal conversion ability	[225]

^1^ Reduced graphene oxide. ^2^ Mesoporous silica nanoparticles. ^3^ Reducible polyethyleneimine passivated carbon dots.

## Data Availability

This article’s data sharing is not applicable as no new data were created or analyzed in this study.

## Abbreviations

aniline functionalized graphene quantum dots	a-GQDs
aptameric functionalized materials	AFMs
carbon nanotubes	CNTs
carbon quantum dots	CQDs
cationic β-cyclodextrin-based polymer	CCD/P
docetaxel-loaded liposomes functionalized with transferrin	LIP-DTX-TF
dopamine	DOPA
dopamine-polyethylene glycol-carboxylic acid	DPA-PEG-COOH
enhanced permeability and retention	EPR
extra-cellular matrix	ECM
folate receptor	FR
folic acid	FA
folic acid-coated gold nanoparticles conjugated with fluorophore	FA-Au-FITC
functionalized nanoparticles	FNPs
gold nanoparticles	AuNPs
graphene oxide	GO
hyaluronic acid	HLA
hydroxyapatite	HDA
lactate dehydrogenase	LDH
poly(lactic-co-glycolic acid)	PLGA
magnetic nanoparticles	MNPs
magnetic resonance imaging	MRI
mesoporous silica nanoparticles	MSN
methacryl polyhedral oligomeric silsesquioxane	M-POSS
nanoparticles	NPs
near-infrared	NIR
nitrogen-doped carbon quantum dots	NCQDs
nitrogen-doped graphene quantum dots	N-GQDs
oleic acid-coated iron oxide NPs	Fe_3_O_4_-OA
phenyl boronic acid	PBA
phenylethylamine	PhEA
photodynamic therapy	PDT
photothermal	PT
piperine	PIP
poly(acrylic acid)	PAA
polyethylene glycol	PEG
polyethylene glycol with thiol end group	thiol-PEG
polyethylene glycol-gelatin-chitosan-hyaluronidase-5-fluorouracil	CS-HYL-5-FU-PEG-G
positron emission tomography	PET
quantum dots	QDs
receptor-mediated endocytosis	RME
reticulum endothelial system	RES
sodium alginate (SA)–polyvinyl alcohol (PVA)–bovin serum albumin	SA-PVA-BSA
sodium alginate/polyethylene glycol (vinyl alcohol)	SA/PVA/Ca
superparamagnetic iron oxide NPs	SPIONs
tetraethylorthosilicate	TEOS
ultra-small superparamagnetic iron oxide NP	USPIONs
